# BioSTEM: A modern educational tool for research and innovation in the field of molecular biology and personalized medicine

**DOI:** 10.1186/s40246-025-00786-x

**Published:** 2025-07-14

**Authors:** George P. Patrinos, Stavroula Siamoglou, Konstantinos Lazaros, Vasilios Xirafas, Aristidis G. Vrahatis, Christina Mitropoulou

**Affiliations:** 1 School of Health Sciences, Department of Pharmacy, Laboratory of Pharmacogenomics and Individualized Therapy, University of Patras, Patras, Greece; 2 Hellenic Pasteur Institute, Athens, Greece; 3 Faculty of Medicine and Health Sciences, Department of Pathology, Clinical Bioinformatics Unit, Erasmus University Medical Center, Rotterdam, The Netherlands; 4 College of Medicine and Health Sciences, Department of Genetics and Genomics, United Arab Emirates University, Al-Ain, Abu Dhabi, UAE; 5 Zayed Center for Health Sciences, United Arab Emirates University, Al- Ain, Abu Dhabi, UAE; 6 Department of Informatics, Ionian University, Corfu, Greece; 7 The Golden Helix Foundation, London, UK

**Keywords:** STEM, BioSTEM, Molecular biology, Genetics, Personalized medicine, Science communication, High school, Students, Educators, Educational and research tool

## Abstract

Personalized Medicine has the potential to transform modern healthcare by tailoring medical decisions to an individual’s unique genomic profile. However, its integration into routine clinical practice is hindered by limited genomics education among healthcare professionals and a general lack of public awareness. To address these challenges, we introduce the BioSTEM initiative, an innovative educational tool designed for primary, elementary, and high school students. BioSTEM combines optimized online learning modules with interactive audiovisual content, fostering direct communication between students and educators. It also integrates alternative learning strategies, such as online games and comics, to enhance engagement with topics in Biology, Molecular Biology, Genetics, and Personalized Medicine. Since 2016, BioSTEM has reached over 6,000 students across three countries through a combination of theoretical instruction and hands-on experiments using a portable molecular biology laboratory. Our results indicate that participation in BioSTEM significantly improved students’ and educators’ perception of their understanding of concepts in Genetics and Personalized Medicine. Participants reported a stronger grasp of genomic concepts and expressed support for incorporating BioSTEM into the standard biology curriculum. These findings suggest that BioSTEM is a promising and scalable approach to advancing biomedical education and fostering the development of genomic-literate citizens.

**Supplementary Information:**

The online version contains supplementary material available at 10.1186/s40246-025-00786-x.

## Introduction

Personalized (or Individualized or Precision) Medicine denotes the optimization of medical decision-making process based on a person’s unique genomic information. Personalized Medicine (PM) aims to enable healthcare professionals to ascertain disease risk, to individualize drug treatment towards improving patients’ quality of life and minimizing the risk of developing adverse reactions, while a number of monogenic diseases and cancers can now be readily identified by distinct variant genotypes and/or gene expression patterns [1, 2]. Contemporary medical genetics and genomics are exceedingly complicated, more so than was genetics at earlier periods of its development, such that it has become very difficult to achieve a sufficient level of understanding as far as healthcare professionals and the general public is concerned. In general, oversimplified views of the subject matter remain widespread among healthcare professionals, while, in the general public there is a tendency to believe that a well-documented family/patient history can reveal as much prevention- and therapy-relevant knowledge as genetic testing, especially for complex traits, such as diabetes, cardiovascular disorders, various types of cancer, etc. Individual choice to be genetically tested may also vary when there are limited or no treatment options [3].

Despite its potential, incorporation of PM interventions into the mainstream medical practice is still in their infancy, due to a number of bottlenecks that have yet to be fully addressed [4]. Apart of the main limiting factors of PM which relate to the various aspects of the science itself, such as incomplete, or in certain cases lack of, knowledge of relevant genotype-phenotype correlations, particularly for polygenic inherited disorders (e.g. autism spectrum disorders, cardiovascular disorders, intellectual disability), there are other important societal issues that still hold back the field, such as: (a) insufficient genetics/genomics education of healthcare professionals, and (b) lack of harmonized PM education in different higher education institutions not only within the same country but most importantly between developed and developing countries. The above issues have the following consequences: (a) widespread public misunderstandings of the intricacies of genetics and genomics, and (b) general lack of awareness of the general public about the potential benefits of genetics and genetic testing, that creates ethical issues, often arising from the commercial misuse of genetic testing by some private genetic testing laboratories, which directly negatively impact on the general public by misleading marketing and exaggerated clinical impact. So far, a number of studies in very few countries, such as the United States, the United Kingdom, Finland, Germany, etc., have indicated that the genetics/genomics education of physicians, especially senior physicians, is a cumbersome issue, in particular in light of the need of applying genomic tools to an increasingly larger number of medical conditions [5–12]. Often, physicians seem to have a relatively poor perception of PM and its potential to adjust conventional medical interventions to the patient’s unique variome. This does not only affect their basic knowledge of genetics but, most importantly, their ability to interpret genetic testing results and utilize them in a clinical setting. This fact has been highlighted not only by a professed lack of genetics/genomics education by mostly the older generation of physicians [5], but also by the comparatively small number of patient referrals to genetic testing laboratories from their side. Furthermore, the general public typically has a comparatively poor understanding of genetics, genomics and its impact on society [5, 6], which does not allow them to fully appreciate the benefits and limitations of genetic testing within the context of PM or to discriminate between scientifically sound and flawed genetic testing services. Moreover, it is necessary to adequately inform and educate respective parties and other interested stakeholders where necessary, in cases in which surveys of the general public’s perceptions or of the opinions of healthcare professionals show the existence of gaps in relevant knowledge and/or misunderstandings of key concepts of PM [13]. From the above, it is clear that although genomics education of future healthcare professionals takes place in the Universities at the undergraduate and post-graduate level, it is imperative to boost genomics awareness of the general public as early as the school years.

The STEM approach (Science, Technology, Engineering and Mathematics) marks the shift from teacher-centered to a student-centered approach based on research and experimentation [14, 15]. According to VanTassel-Baska and Little (2011), this type of pedagogical approach is the best practice for all, as it will lead to the development of the student’s skills in science, technology, engineering and mathematics [16]. Also, Moore and coworkers (2014) mentioned the six principles for a quality STEM education: (a) integrating different scientific fields into the school curriculum, (b) a student-centered pedagogical approach, (c) motivating the student, (d) redesigning, (e) make mistakes and learn from them, and (f) focus on teamwork [17].

Here, we present BioSTEM, an innovative educational initiative that aims to address the major educational challenges and bottlenecks outlined above, aiming to enrich knowledge among primary, elementary and high-school students in the fields of molecular biology, genetics and PM and to contribute towards expanding genomics awareness among future citizens.

## Methods

### Description of the biostem educational tool

The BioSTEM concept includes two separate modules, namely (a) Teaching scenarios, and (b) Laboratory practices. These are available at the BioSTEM portal (www.biostem.gr; content in Greek; Supplementary Fig. 1).

Teaching scenarios aim to enrich students’ knowledge in the fields of Biology, Molecular Biology and Genetics in an innovative fashion. These scenarios are delivered out-of-school and are designed within the scope of asynchronous learning. Each scenario is designed to be tightly linked to the official school textbook and curriculum and includes prior required knowledge and description of the aims and goals, followed by detailed educational material, hyperlinks to corresponding literature and accompanied by questions to evaluate the students. The teaching scenarios are dynamic and can be updated regularly, while novel teaching scenarios can be added to the BioSTEM portal to enrich the existing teaching material. Although the teaching scenarios are linked to the official school textbook, they are not yet formally embedded to the school curriculum.

Laboratory practices include hands-on training in the field of Molecular Biology and Genetics. They are designed such to expand theoretical knowledge with a hands-on training, allowing a more direct engagement of the students, hence aiming to a more in-depth understanding of the topics learned in the corresponding teaching scenarios. Laboratory practices are performed using a portable Molecular Biology laboratory that can be carried in a small suitcase, even in schools in remote areas. All students complete the same experimental activities but using an adjusted grade-appropriate language from the instructors. Students are also asked to interpret data, resulting from the DNA analysis and to delineate these with a specific phenotype (please see below). These activities are carried out by high school teachers, who can be also trained, using a dedicated 3-hour long train-the-trainer session, to deliver these in a more interactive fashion.

Additional training and educational sessions in the laboratory practices for primary school students include the construction of double-stranded DNA with plasticine, and a dedicated comics series and online games that describe in simple terms concepts which are related to the Molecular Biology and Genetics.

### Experimental procedures

**Fig. 1:**
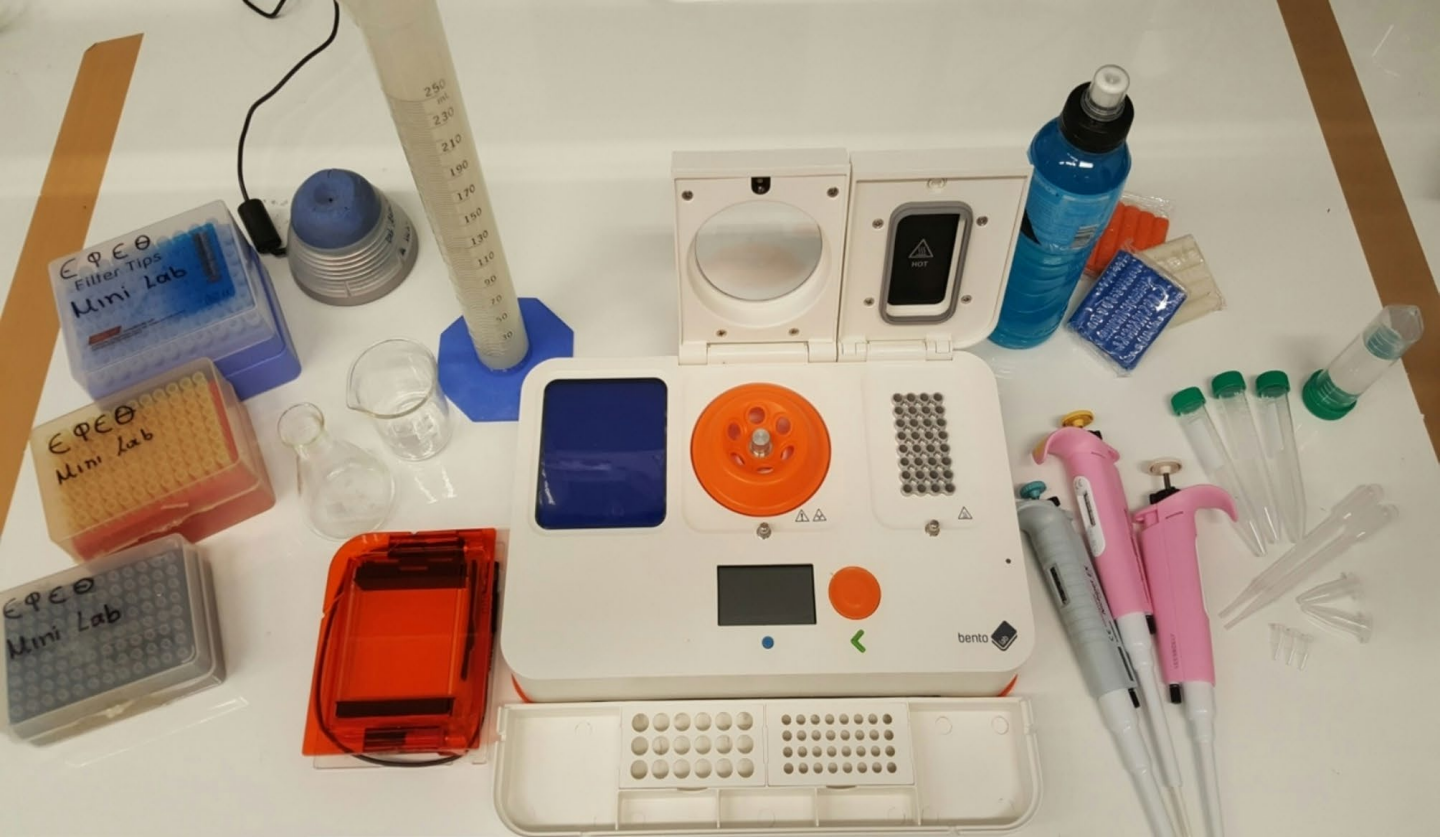
The portable Molecular Biology laboratory. The device includes a built-in thermal cycler, a centrifuge, an electrophoresis device, and a UV transilluminator. The experimental setting comes with glass- and plasticware, adjustable pipettes, a vortex and a heater. The entire setting is well-designed for easy transport in a small suitcase

Experimental procedures are run on the Bento Lab (www.bento.bio) portable molecular biology laboratory that includes: a 32-well thermal cycler, a 13 K rpm centrifuge, an electrophoresis device, coupled to an ultraviolet transilluminator (Fig. 1). The experimental setting also includes a vortex, a heater, glass- and plasticware and all consumables and reagents required for the conduct of the experiments.

DNA extraction is performed from epithelial cells of the inner cheek surface using an isotonic commercial beverage [18]. The extracted DNA is subsequently used in polymerase chain reaction experiments to amplify a *TAS2R38* gene fragment that includes the rs1726866T/C variant, which correlates with the perception of the bitter taste of phenylthiocarbamide (PTC). The variant form of this SNP results in the replacement of a Valine (V) to Alanine (A) in the protein sequence. More specifically, Valine appears in people who do not perceive the bitter taste (non-tasters) of the PTC compound, while Alanine appears in people who perceive it (tasters). In this phase, students are asked to taste cabbage and then answer the question “How do you taste cabbage?” by grading the bitter taste that a student perceives, from 1 to 3 (1 indicates mild, 2 indicates moderate and 3 indicates strongly bitter taste). It is emphasized that the different perception of the bitter taste is the result of a combination of at least 3 genetic variants in the flavor receptor family, and as such, the result is likely to deviate from the expected genotype-phenotype correlation in the context of this educational activity, which focuses only on a single gene variant. However, it should be noted that this activity is educational rather than diagnostic, so any possible deviation did not pose a problem for the final educational goal. The final PCR product was subsequently digested with the restriction enzyme *Fnu4H1* and visualized in gel electrophoresis.

### Student questionnaires

In order to assess the impact of the BioSTEM educational tool in raising genomics awareness and literacy among students and teachers, we have compiled and distributed questionnaires to assess the level of genomics knowledge, distributed physically in hard-copies to the participants, before and after a BioSTEM session. There are dedicated questionnaires available for students and educators. In particular, students’ questionnaires included 13 questions, split in 3 parts, aiming to (a) assess the opinion of students about the biology course (Part A), (b) evaluate the knowledge of the students on basic concepts in Biology and Genetics (Part B), and (c) to inquire about the possible impact of BioSTEM onto the main school educational process (Part C). Also, the educators’ questionnaires included 17 questions, again split in 3 parts, aiming to (a) assess their opinion on how much the students engaged in the biology course (Part A), (b) evaluate their knowledge on basic concepts in Biology and Genetics (Part B), and (c) to inquire about the possible impact of BioSTEM onto the main school educational process (Part C). Separate questionnaires were distributed before and after a BioSTEM session, both for students (Supplementary Material S1 and S2) and educators (Supplementary Material S3 and S4). The study was approved by the University of Patras Bioethics Committee.

### Statistical analysis

Statistical analysis was conducted to evaluate the impact of the BioSTEM on students’ and educators’ understanding of key concepts in Molecular Biology, Genetics, and Personalized Medicine. To assess the statistical significance of differences in comprehension before and after participation in BioSTEM sessions, questionnaire data were analyzed using established non-parametric statistical methods. A series of box plots were employed to visualize the distribution of responses before and after BioSTEM interventions, providing an intuitive representation of potential shifts in understanding. To formally infer statistical significance, the Mann-Whitney U test was applied, a robust non-parametric test suitable for comparing independent samples that do not necessarily follow a normal distribution. The significance threshold was set at p-values < 0.05, with results interpreted accordingly. In this context, p-values below 0.05 were deemed statistically significant, indicating a meaningful difference in participants’ conceptual grasp following the BioSTEM sessions.

To enhance the clarity of the statistical findings, the box plots were annotated with significance markers. Specifically, asterisks were assigned to indicate varying levels of significance, with “****” denoting p-values < 0.0001, “***” denoting p-values < 0.001, “**” corresponding to p-values < 0.01, and “*” representing p-values < 0.05. Non-significant results were labeled as “ns” to denote the absence of statistically meaningful differences. The analysis was implemented using the Python programming language, leveraging the scipy library for statistical testing and matplotlib for visualization.

## Results

### Content of the biostem portal

The content of the BioSTEM portal (www.biostem.gr) is currently only available in the Greek language. Initial topics of the teaching scenarios, compiled by the BioSTEM affiliated educators, included: (a) Physical sciences, (b) Bioethics, (c) Ethics in Genetics, (d) Evaluating scientific articles, (e) History of Science, (f) Biology of Nature, (g) Laboratory rules, (h) The cell, (i) Diseases, Medicine and Drugs, and (j) Molecular Biology and Genetics. Also, the experimental procedures presented to students were: (a) DNA isolation, (b) DNA amplification using Polymerase Chain Reaction (PCR), (c) DNA electrophoresis and (d) Digestion of DNA fragments using restriction enzymes. These scenarios were further updated, while new ones are currently being compiled and expected to be added for the next school year.

### BioSTEM sessions

**Fig. 2:**
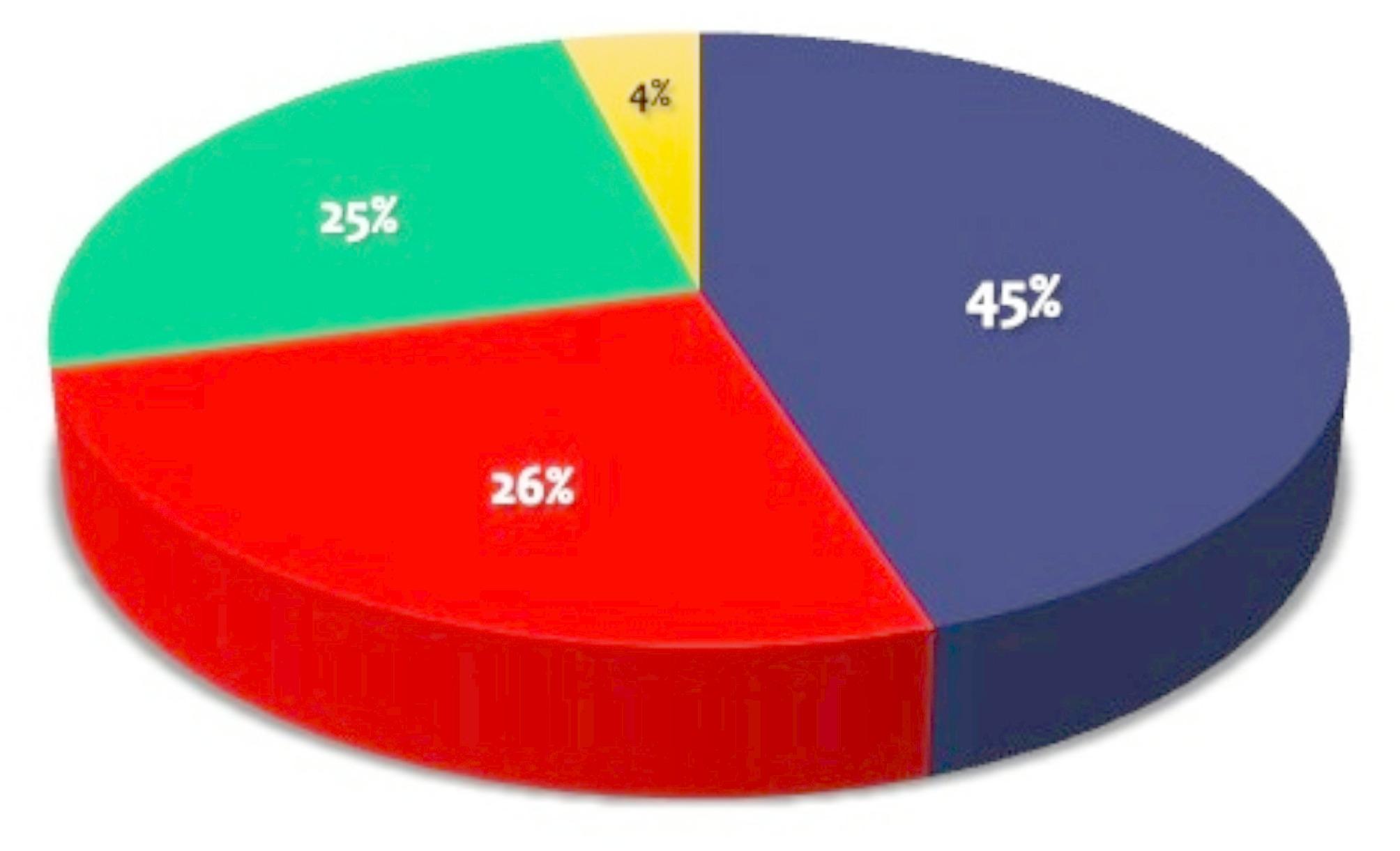
Summary statistics from the various BioSTEM educational activities with the portable molecular biology laboratory (2018–2024). The majority includes students over 16 year (45%; blue), followed by students between 12–16 year (26%; red) and students below 12 year (25%; green). 4% included educators and school teachers (in yellow)

Since the beginning of BioSTEM in 2016, more than 6000 school students have been trained in the following occasions: (a) 38 high schools in 22 cities in Greece, where primary, elementary and high school students have participated (Fig. 2), (b) 5 Science Festivals in Athens (2019, 2022, 2023, 2024) and Patras (2018), where Molecular Biology experiments are presented not only to students and teachers but also to their custodians and numerous attendees from the general public, (c) the European Commission-funded “Researchers’ Night” activities in 2017, 2022, 2023 and 2024. (d) 2 National (Greek National Personalized Medicine Conferences; 2021, 2023) and 4 international conferences in Greece (European Pharmacy Student Association, 2022) and abroad [23rd Golden Helix Pharmacogenomics Day, Al-Ain, UAE (2020), BioSTEM Conference, Bucharest, Romania (2023), and 2024 Precision Medicine Expo and Summit, Dubai, UAE (2024)]. Also, 50 school teachers and educators got trained and actively involved in these activities, using the train-the-trainers approach. In all these occasions, short presentations before the experimental procedures explained both the experiments as well as the expected results and their interpretation in an understandable fashion.

### Questionnaires

In order to better measure the impact of the BioSTEM activities, dedicated questionnaires were distributed to school students and teachers and educators before (459 students and 19 teachers) and after (501 students and 20 teachers) each BioSTEM session (Table 1, Supplementary Material S1-S4, Supplementary Figs. 1–3). Our data show that students have better understood the notions of Biology and Molecular Biology (Q1, Q2; *p* < 0.0001), got intrigued by the notions of Molecular Biology and Genetics (Q4; *p* < 0.0001) and would be interested in getting involved with Molecular Biology and Genetics in the future (Q5; *p* < 0.01). Also, students reported an improvement of their understanding of the correlation between genomic variants and the predisposition to a genetic disease (Q6; *p* < 0.0001), the correlation between genomic variants and response to drugs (Q7; *p* < 0.0001) and the overall notion of Pharmacogenomics (Q8; *p* < 0.0001). Lastly, the students felt that a BioSTEM session could ideally accompany the biology courses in their school curricula (Q13; *p* < 0.01). We failed to observe any statistically significant differences among different school ages, as the sample size when splitting for different ages was rather small.

**Table 1:** Demographic information of the respondents of the students and educators’ questionnaires

Students (22 schools)	Before – *n* (%)	After – *n* (%)
**Boys**	164 (35.73)	172 (34.33)
**Girls**	279 (60.78)	306 (61.08)
**Not answered**	17 (3.49)	23 (4.59)
**Total**	459 (100.00)	501 (100.00)
**Teachers / Educators (8 schools)**	**Before – n (%)**	**After – n (%)**
**Male**	7 (36.84)	8 (40.00)
**Female**	12 (63.16)	12 (60.00)
**Total**	19 (100.00)	20 (100.00)

**Fig. 3:**
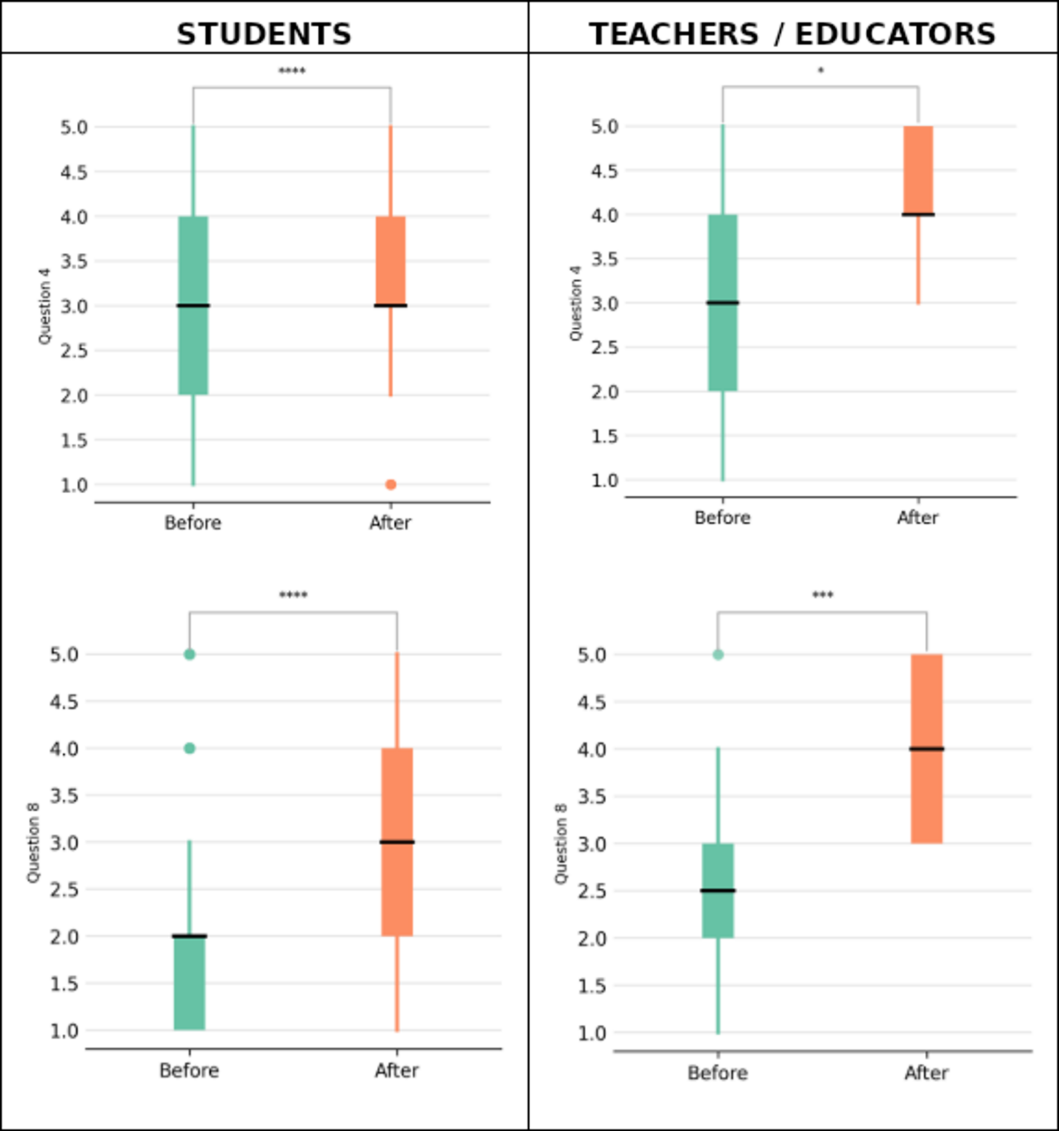
Box plots illustrating the distribution of responses before and after the BioSTEM educational intervention for both students and educators. The left column represents students’ responses, while the right column corresponds to educators’ responses. The first row depicts responses to Question 4, which assesses participants’ level of interest and engagement in Molecular Biology and Genetics, whereas the second row presents responses to Question 8, which evaluates their awareness and understanding of Pharmacogenomics and Personalized Medicine. A Mann-Whitney U test was performed to determine statistical significance, with significance levels indicated by asterisks: * (*p* < 0.05), *** (*p* < 0.001), and **** (*p* < 0.0001). The results demonstrate a significant improvement in comprehension and awareness among both students and educators following participation in BioSTEM sessions

School teachers and educators reported that the BioSTEM session significantly enhanced their understanding of key concepts in Molecular Biology and Genetics (Q4; *p* < 0.05). They also indicated increased awareness of topics such as Pharmacogenomics and Precision Therapeutics (Q8; *p* < 0.001). Furthermore, they expressed confidence that their students could grasp the relationship between drug dose adjustment and a patient’s genetic profile (Q10; *p* < 0.05), as well as accurately describe these terms (Q11; *p* < 0.01; Supplementary Figs. 4–6). Interestingly, data from the questionnaires analysis indicated that both students and teachers / educators believed that the BioSTEM can spark the students’ interest in topics related to Molecular Biology and Genetics (Q4) and made them aware of the notions of Pharmacogenomics and Personalized Therapy such that they can explain these notions in their social circle (Q8; Fig. 3).

**Fig. 4:**
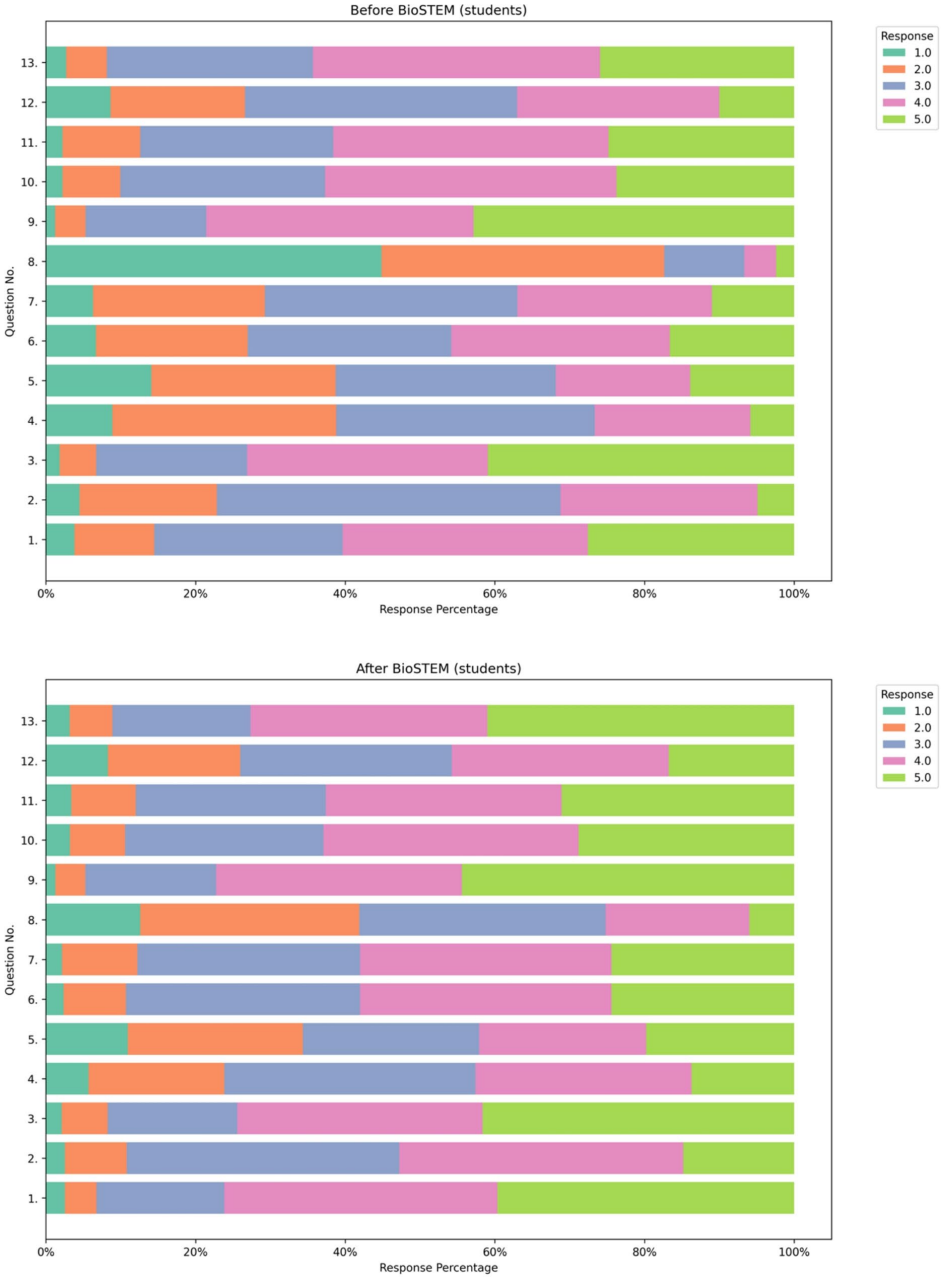
Diverging stacked bar plots illustrating the distribution of student responses to 13 questionnaire items administered before (left) and after (right) participation in BioSTEM sessions. Responses are color-coded on a Likert scale from 1.0 (lowest agreement or understanding) to 5.0 (highest). Following the educational intervention, a clear shift toward higher response values (4.0 and 5.0) is observed across nearly all questions, indicating a marked improvement in student understanding, engagement, and awareness of topics related to Molecular Biology, Genetics, and Personalized Medicine

**Fig. 5:**
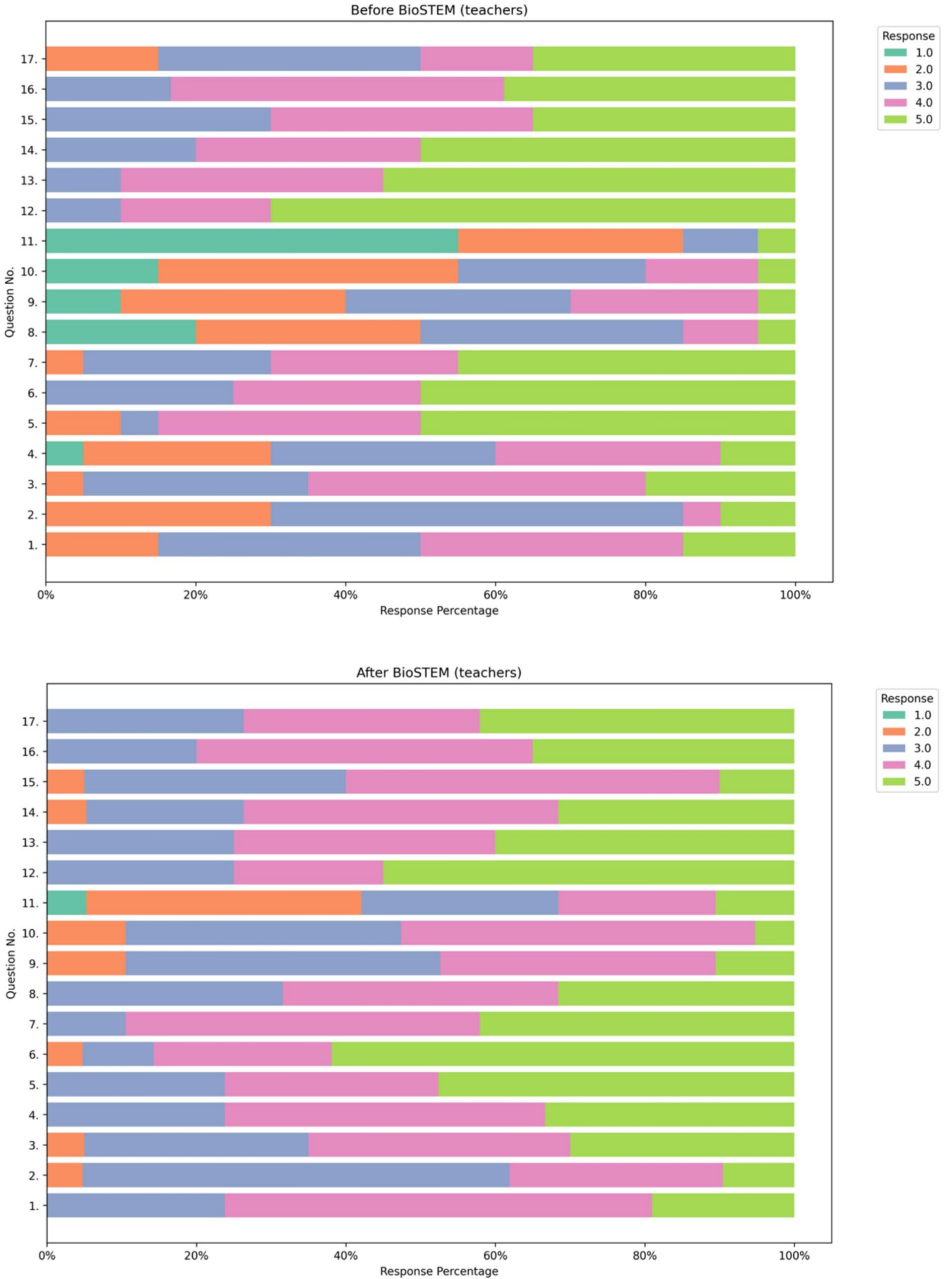
Diverging stacked bar plots showing the distribution of teacher and educator responses to 17 questionnaire items collected before (left) and after (right) participation in BioSTEM sessions. Responses are displayed using a Likert scale ranging from 1.0 (lowest agreement or understanding) to 5.0 (highest). After the BioSTEM intervention, there is a clear overall increase in higher-level responses (4.0 and 5.0), indicating improved understanding of core concepts such as Molecular Biology, Genetics, Pharmacogenomics, and their relevance in school education. These results suggest that BioSTEM also positively influences educators’ perceptions of genomics education and its integration into teaching practice

This positive impact of the BioSTEM sessions is further supported by the graphical analysis of the questionnaire responses. As illustrated in Fig. 4, student responses before and after the intervention reveal a distinct shift toward stronger agreement and perceptions of understanding across nearly all questionnaire items, underscoring an overall improvement in comprehension, engagement, and familiarity with key topics in Molecular Biology, Genetics, and Personalized Medicine. A similar trend is evident among teachers and educators (Fig. 5), where post-intervention responses indicate enhanced perceptions of understanding and appreciation of core concepts, particularly regarding the integration of Genetics and Pharmacogenomics/Personalized Medicine into school education. These findings further highlight the educational value of BioSTEM in fostering both knowledge and positive attitudes toward genomics-related topics.

Our results show that the BioSTEM sessions have contributed towards a better understanding of the notions of Molecular Biology, Genetics and Personalized Medicine and Therapeutics both to students and educators.

## Discussion

A fundamental aspect to expedite clinical implementation of personalized medicine is raising genomics awareness of the general public. Although genomics education of future healthcare professionals takes place in the Universities at the undergraduate and post-graduate level, we conceived and implemented the BioSTEM concept to raise genomics awareness of the general public as early as the school years.

BioSTEM represents a scalable and adaptable educational model that can be seamlessly integrated into diverse national curricula. By combining localized content, initially developed in Greek, with universally relevant scientific concepts, the initiative offers a flexible framework that can be translated and implemented across different linguistic and cultural contexts. At the same time, BioSTEM bridges the longstanding gap between secondary education and the biomedical research community. By introducing students to hands-on molecular biology techniques and emerging fields such as pharmacogenomics, it brings cutting-edge science into the classroom, nurturing early interest in genomic research and promoting deeper scientific literacy from a young age. Also, it engages students in Scientific Practices, which also constitutes a critical part of United States Next Generation Science Standards (https://www.nextgenscience.org).

Interestingly, the BioSTEM concept also indirectly involves parents and custodians. In particular, the BioSTEM concept aims to (a) disseminating the knowledge and the research practice in Molecular Biology and Genetics to biology courses in the high school education (b) enhancing the research school activities with interactive and hands-on training on the portable molecular biology laboratory, and (c) increase public awareness about the importance of the analysis of the human genome and the benefits of personalized medicine into clinical practice. The main purpose is to motivate students through their school practice and increase their interest for the emerging disciplines of Molecular Biology, Genetics and Personalized Medicine.

Our BioSTEM approach has taken into consideration the challenges of implementing the STEM approach in secondary high school education [19], as well as the lack of inter-relationships between schools and the University. Specifically, BioSTEM attempts to exploit the STEM approach to migrate the concepts of Molecular Biology, Genetics and Personalized Medicine from the University to the high school curriculum. BioSTEM clearly combines active learning jointly with case-based learning in elementary and high school, an approach that is usually employed in universities and very rarely during high school years [20].

The BioSTEM was designed as an innovative educational tool, accessible online, which includes online learning modules about various aspects of Biology, accompanied by additional audiovisual supportive material, a forum that allows direct communication between the users and the BioSTEM team. BioSTEM sessions are often accompanied by online games and plasticine craftsmanship sessions, especially designed for primary school students.

The inclusion of practical training sessions with the portable molecular biology laboratory upgraded the overall training experience and expanded genomics education among participants. Responses from the various questionnaires both from students and educators indicate that they perceive an increase in their understanding of notions like disease prevention, genome-guided therapy, genetic diseases, and so on. Furthermore, BioSTEM emphasized the bioethical issues arising from the analysis of the human genome, positively impact on the students, their parents and custodians.

Once the BioSTEM approach gets fully integrated into the educational process and school curricula, it would enable the fields of Biology, especially Molecular Biology and Genetics to grow at a faster pace. A similar approach has been proposed by the International Committee on Clinical Molecular Biology Curriculum of the International Federation of Clinical Chemistry for enhancing healthcare professionals genetic testing skills, but not as early as school years and using a far more complicated and difficult to transport platform [21].

Finally, the BioSTEM concept, would be particularly useful for remote areas, especially the portable molecular biology laboratory. The easy-to-transport equipment would not only be applicable to genomics education but also as an attractive solution to provide a portable instrumentation for low-throughput genetic and pharmacogenomics analysis in remote areas without any central Molecular Biology laboratory facilities. With such a “plug-and-play” instrument, molecular genetic testing can be more broadly implemented and made available to a much bigger patient community, as a point-of-care molecular diagnostic solution. This has already been demonstrated in the field of personalized treatment [22].

To conclude, the BioSTEM educational approach using a portable Molecular Biology laboratory can hold promise for an innovative interactive educational tool for Molecular Biology and Genetics that can be used to raise genomics awareness of the general public, ideally starting from school age. The adoption of this innovative approach from the large-scale pan-European Genome of Europe genomics project will likely contribute to raising genomics awareness in all 27 participating countries across Europe.

What sets BioSTEM apart is its strong foundation in evidence-based evaluation. The program has demonstrated statistically significant improvements in student and educator understanding and engagement, as measured through pre- and post-session questionnaires. This data-driven approach supports BioSTEM’s educational efficacy and distinguishes it from other STEM initiatives that often rely on qualitative or anecdotal feedback. Moreover, the use of a portable and cost-effective laboratory setup ensures that BioSTEM is accessible to students even in remote or underserved regions. By decentralizing access to practical genomics education, the program not only promotes educational equity but also lays the groundwork for broader and more inclusive implementation of personalized medicine in future healthcare systems.

## Electronic supplementary material

Below is the link to the electronic supplementary material.

Supplementary Material 1

## Data Availability

No datasets were generated or analysed during the current study.

## References

[1] Cooper DN, et al. Genes, mutations, and human inherited disease at the dawn of the age of personalized genomics. Hum Mutat. 2010;31(6):631–55. 20506564 10.1002/humu.21260

[2] Guttmacher AE, et al. Personalized genomic information: Preparing for the future of genetic medicine. Nat Rev Genet. 2010;11(2):161–5. 20065954 10.1038/nrg2735

[3] Patenaude AF. Genetic testing and psychology. In Molecular Diagnostics (2nd ed.) Patrinos GP, Ansorge WJ, editors. Elsevier/Academic Press, pg. 549–562 (2009).

[4] Kampourakis K, et al. Key challenges for next generation pharmacogenomics. EMBO Rep. 2014;15(5):472–6. 24723683 10.1002/embr.201438641 PMC4210086

[5] Mai Y, et al. A critical view of the general public’s awareness and physicians’ opinion of the trends and potential pitfalls of genetic testing in Greece. Per Med. 2011;8(5):551–61. 29793257 10.2217/pme.11.48

[6] Mai Y, et al. Critical appraisal of the views of healthcare professionals with respect to pharmacogenomics and personalized medicine in Greece. Per Med. 2014;11(1):15–26. 29751393 10.2217/pme.13.92

[7] Makeeva​‌ OA, et al. An epidemiologic-based survey of public attitudes towards predictive genetic testing in Russia. Per Med. 2010;7(3):291–300. 29776224 10.2217/pme.10.23

[8] Hietala M, et al. Attitudes toward genetic testing among the general population and relatives of patients with a severe genetic disease: a survey from Finland. Am J Hum Genet. 1995;56(6):1493–500. 7762573 PMC1801087

[9] Balck F, et al. Attitudes toward genetic testing in a German population. Genet Test Mol Biomarkers. 2009;13(6):743–50. 19810825 10.1089/gtmb.2008.0154

[10] Cherkas LF, et al. A survey of UK public interest in internet-based personal genome testing. PLoS ONE. 2010;5(10):e13473. 20976053 10.1371/journal.pone.0013473 PMC2957412

[11] Goddard KA, et al. Public awareness and use of direct-to-consumer genetic tests: results from 3 state population-based surveys, 2006. Am J Public Health. 2009;99(3):442–5. 19106425 10.2105/AJPH.2007.131631 PMC2661444

[12] Ries NM, et al. Willingness to pay for genetic testing: a study of attitudes in a Canadian population. Public Health Genomics. 2010;13(5):292–300. 19864872 10.1159/000253120

[13] Mitropoulou C, et al. Documentation and analysis of the policy environment and key stakeholders in pharmacogenomics and genomic medicine in Greece. Public Health Genomics. 2014;17(5–6):280–6. 25228172 10.1159/000365896

[14] Odom AL, Bell CV. Associations of middle school student science achievement and attitudes and science with student-reported frequency of teacher lecture demonstrations and student-centered learning. Int J Environ Sci Educ. 2015;10(1):87–97.

[15] Tamim SR, Grant MM. Definitions and uses: case study of teachers implementing project-based learning. Interdisciplinary J Problem-Based Learn. 2013;7(2):72–101.

[16] VanTassel-Baska J, Little CA. Content-based curriculum for high-ability learners. Waco, TX: Prufrock; 2011.

[17] Moore TJ, Stohlmann MS, Wang HH, Tank KM, Glancy A, Roehrig GH. Implementation and integration of engineering in K-12 STEM education. In: Strobel J, Purzer S, Cardella M, editors. Engineering in precollege settings: research into practice. Rotterdam: Sense; 2014.

[18] Hearn RP, Arblaster KE. DNA extraction techniques for use in education. Biochem Mol Biol Educ. 2010;38:161–6. 21567818 10.1002/bmb.20351

[19] MacFarlane B. Infrastructure of comprehensive STEM programming for advanced learners. In: MacFarlane B, editor. STEM education for High-Ability learners designing and implementing programming. Waco, TX: Prufrock; 2016. pp. 139–60.

[20] Moro C, Phelps C. Encouraging study in health sciences: informing school students through interprofessional healthcare simulations. Simul Healthc. 2024;19:144–50. 37255339 10.1097/SIH.0000000000000732

[21] Lianidou E, Ahmad-Nejad P, Ferreira-Gonzalez A, Izuhara K, Cremonesi L, Schroeder ME, Richter K, Ferrari M, Neumaier M. Advancing the education in molecular diagnostics: the IFCC-Initiative clinical molecular biology curriculum (C-CMBC); a ten-year experience. Clin Chim Acta. 2014;436:5–8. 24815033 10.1016/j.cca.2014.04.031

[22] Psarias G, Iliopoulou E, Liopetas I, Tsironi A, Spanos D, Tsikrika A, Kalafatis K, Tarousi D, Varitis G, Koromina M, Siamoglou S, Patrinos GP. Development of rapid Pharmacogenomic testing assay in a mobile molecular biology laboratory (2MoBiL). OMICS. 2020;24:660–6. 33064577 10.1089/omi.2020.0168

