# Supplementary material for: BioSTEM: A modern educational tool for research and innovation in the field of molecular biology and personalized medicine
Source: Hum Genomics. 2025 Jul 14;19:82. doi: 10.1186/s40246-025-00786-x (PMC12261724; doi:10.1186/s40246-025-00786-x)

BioSTEM: A modern educational tool for research and innovation in the field of Molecular Biology and Personalized Medicine

George P, Patrinos[1,2,3,4,5,*], Stavroula Siamoglou[1], Konstantinos Lazaros[6], Vasilios Xirafas[1], Aristidis G. Vrahatis[6], Christina Mitropoulou[7]

[1] University of Patras, School of Health Sciences, Department of Pharmacy, Laboratory of Pharmacogenomics and Individualized Therapy, Patras, Greece

[2] Hellenic Pasteur Institute, Athens, Greece

[3] Erasmus University Medical Center, Faculty of Medicine and Health Sciences, Department of Pathology, Clinical Bioinformatics Unit, Rotterdam, the Netherlands

[4] United Arab Emirates University, College of Medicine and Health Sciences, Department of Genetics and Genomics, Al-Ain, Abu Dhabi, UAE

[5] United Arab Emirates University, Zayed Center for Health Sciences, Al-Ain, Abu Dhabi, UAE

[6] Ionian University, Department of Informatics, Corfu, Greece

[7] The Golden Helix Foundation, London, UK

Supplementary Information

S1. Questionnaires distributed to high school <u>students</u> BEFORE a BioSTEM session

Part A

1A. How much do you like the Biology class?

2A. To what extent do you understand the concept of "Biology" and "Molecular Biology"?

3A. How intrigued are you by the concept of "experiment" and "research"?

4A. To what extent does the school, in the context of teaching Biology, stimulate your interest in topics related to Molecular Biology and Genetics?

5A. How interested would you be in future work in the field of Molecular Biology and Genetics?

Part B

6A. How well do I know that there is a correlation between the occurrence of a disease and the person's genetic makeup?

7A. How well do you know that there is an association between the dose of a drug a patient should receive and their genetic makeup?

8A. How often do you hear the terms Pharmacogenomics and Personalized Therapy from my social environment?

9A. How important do you consider the application of Personalized Medicine in human health?

Part C

10A. Do you think the BioSTEM program will help you familiarize yourself with the fields of Biology, Molecular Biology and Genetics?

11A. Do you think that the educational tools of the BioSTEM program will facilitate your familiarity with this academic subject?

12A. To what extent do you think that the BioSTEM program will contribute to shaping your professional orientation?

13A. To what extent do you think the BioSTEM program could accompany the Biology course at school?

S2. Questionnaires distributed to high school students AFTER a BioSTEM session

Part A

1B. Did you enjoy your further engagement with the Biology course in the BioSTEM program?

2B. Have you understood the concept of "Biology" and "Molecular Biology"?

3B. How much did the BioSTEM program sparked your interest in the concept of "experiment" and "research"?

4B. Do you think that the school, in the context of teaching Biology, sparked your interest in topics related to Molecular Biology and Genetics?

5B. Would you be interested in future work in the field of Molecular Biology and Genetics?

Part B

6B. To what extent did you understand that there is a correlation of the occurrence of a disease with the person's genetic makeup?

7B. To what extent did you understand that there is a correlation between the dose of a drug a patient should receive and their genetic makeup?

8B. To what extent will you now mention the concepts of Pharmacogenomics and Personalized Therapy in your social circle?

9B. How important do you consider the application of Personalized Medicine to human health after your involvement with the BioSTEM program?

Part C

10B. Do you think the BioSTEM program helped you familiarize yourself with the fields of Biology, Molecular Biology and Genetics?

11B. Do you think that the educational tools of the BioSTEM program made it easier for you to familiarize yourself with this academic subject?

12B. To what extent do you think that the BioSTEM program contributed to shaping of your professional orientation?

13B. To what extent do you think the BioSTEM program could accompany the Biology course at school?

S3. Questionnaires distributed to high school teachers BEFORE a BioSTEM session

Part A

1A. To what extent do the students like the biology course?

2A. To what extent do you think the students understand the concept of "Biology" and "Molecular Biology"?

3A. Do you think the students are interested in familiarizing themselves with the concept of "experiment" and "research"?

4A. Do you think that the school, in the context of teaching Biology, sparks the student's interest in topics related to Molecular Biology and Personalized Medicine?

5A. Do you find your involvement with Molecular Biology and Personalized Medicine interesting?

Part B

6A. To what extent do you understand that there is a correlation between the occurrence of a disease and the person's genetic makeup?

7A. To what extent do you understand that there is a correlation between the dose of a drug a patient should receive and their genetic makeup?

8A. How often do you hear concepts related to Pharmacogenomics and Personalized Therapy?

9A. To what extent do you think the students understand that there is a correlation between the occurrence of a disease and the person's genetic makeup?

10A. To what extent do you think the students understand that there is a correlation between the dose of a drug a patient should receive and their genetic background?

11A. How often do you hear students refer to concepts related to Pharmacogenomics and Personalized Therapy?

Part C

12A. As an educator, do you consider the application of Personalized Medicine to human health important?

13A. Do you think the BioSTEM program will help familiarize students with the fields of Biology, Molecular Biology and Genetics?

14A. Do you think that the educational tools of the BioSTEM program will help familiarize students with this subject?

15A. To what extent do you think that the BioSTEM program will contribute to shaping the professional orientation of the students?
16A. To what extent do you think that the BioSTEM program will contribute to shaping the professional orientation of the students?
17A. How excited were your students about Biology before BioSTEM?

S4. Questionnaires distributed to high school teachers AFTER a BioSTEM session

Part A
1B. How much did the students like their further engagement with the biology course in the context of the BioSTEM program?
2B. To what extent do you think the students were familiar with the concept of "experiment" and "research" after the BioSTEM program?
3B. Do you think that the BioSTEM program introduced the students to the concept of "experiment" and "research"?
4B. Do you think that the school in the context of teaching the subject of Biology in combination with the BioSTEM program sparked the student's interest in topics related to Molecular Biology and Genetics?
5B. Did you find your involvement in Molecular Biology and Personalized Medicine interesting within the BioSTEM program?

Part B
6B. To what extent did you understand that there is a correlation between the occurrence of a disease and the person's genetic background?
7B. To what extent did you understand that there is a correlation between the dose of a drug a patient should receive and their genetic background?
8B. How well can you now explain concepts related to Pharmacogenomics and Personalized Therapy to those around you?
9B. To what extent do you think the students understood that there is a correlation between the occurrence of a disease and the person's genetic background?
10B. To what extent do you think the students understood that there is a correlation between the dose of a drug a patient should receive and their genetic background?

11B. How easily do you now hear students refer to concepts related to Pharmacogenomics and Personalized Therapy?

Part C

12B. As an educator, do you consider the application of Personalized Medicine to human health important after BioSTEM?

13B. Do you think the BioSTEM program helped familiarize students with the fields of Biology, Molecular Biology and Genetics?

14B. Do you think that the educational tools of the BioSTEM program helped to familiarize the students with this academic subject?

15B. To what extent do you think the BioSTEM program helped shapiung the students' career orientation?

16B. To what extent do you think the BioSTEM program could accompany the Biology course at school?

17B. How excited are your students about Biology after BioSTEM?

Supplementary Figure 1

Comparison between the students' responses in the Part A questions of the student's questionnaires before and after the BioSTEM session. ns: Not significant, **: p-value<0.01, ****: p-value<0.0001.

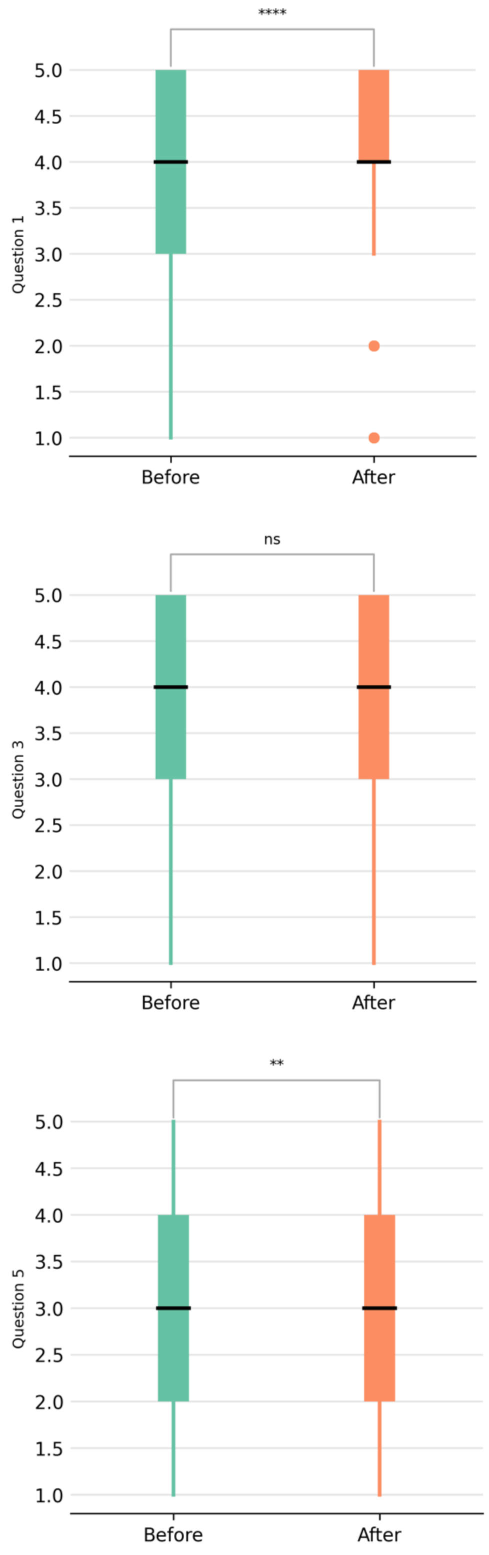

Supplementary Figure 2

Comparison between the students' responses in the Part B questions of the student's questionnaires before and after the BioSTEM session. ns: Not significant, ****: p-value<0.0001.

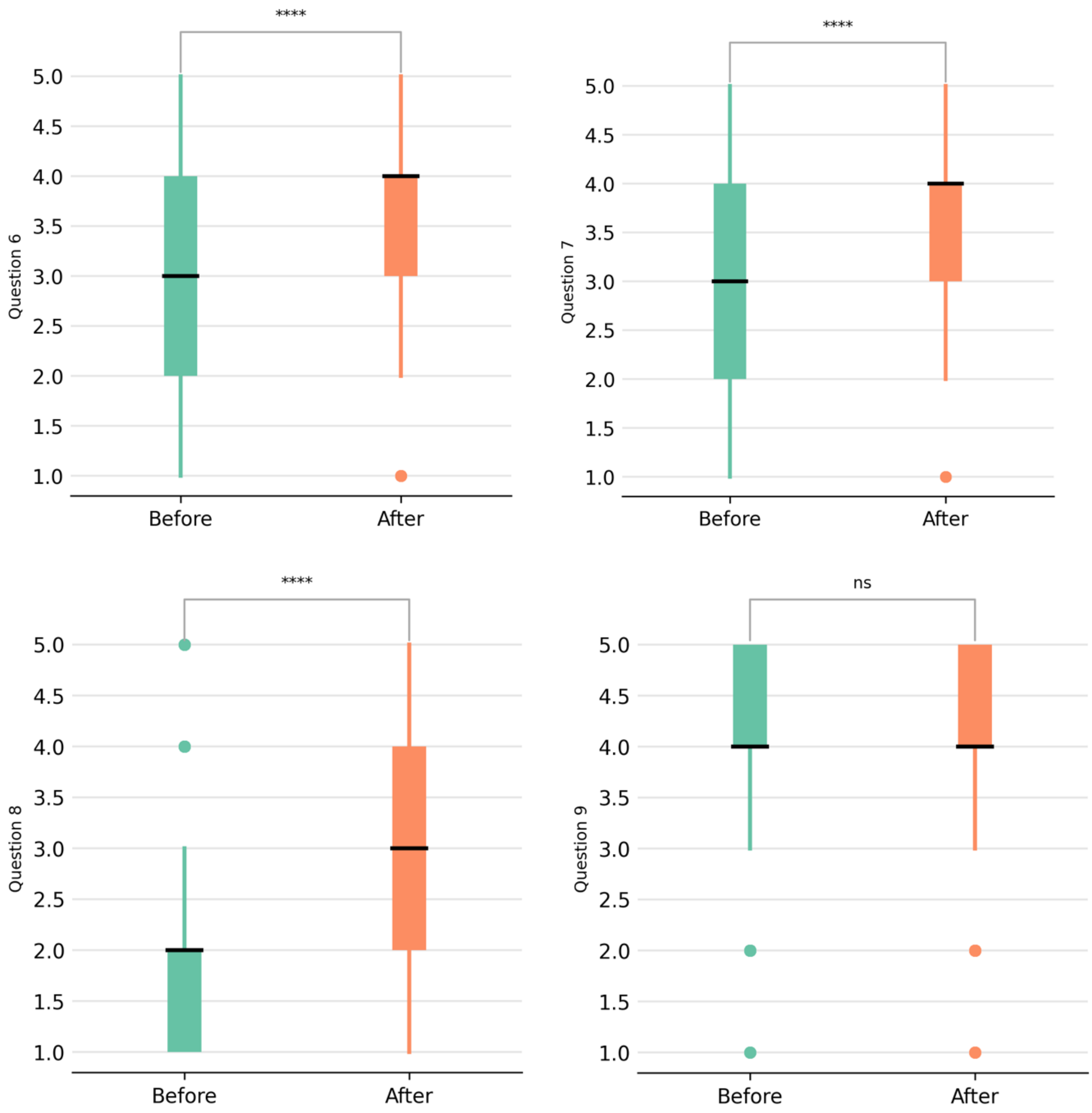

Supplementary Figure 3

Comparison between the students' responses in the Part C questions of the student's questionnaires before and after the BioSTEM session. ns: Not significant, ***: p-value<0.001.

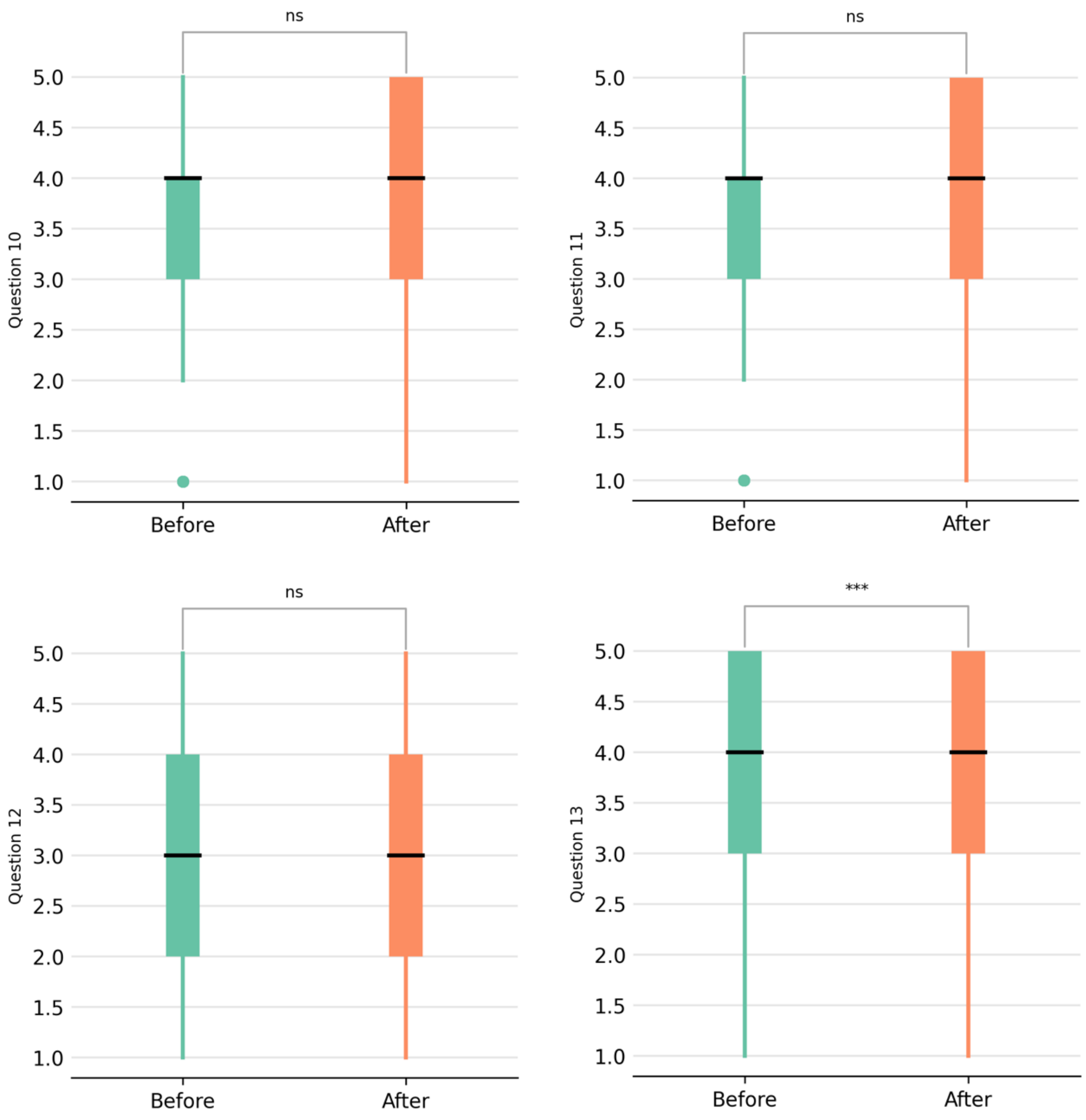

Supplementary Figure 4

Comparison between the teachers’ responses in the Part A questions of the teacher’s questionnaires before and after the BioSTEM session. ns: Not significant, *: p-value<0.05.

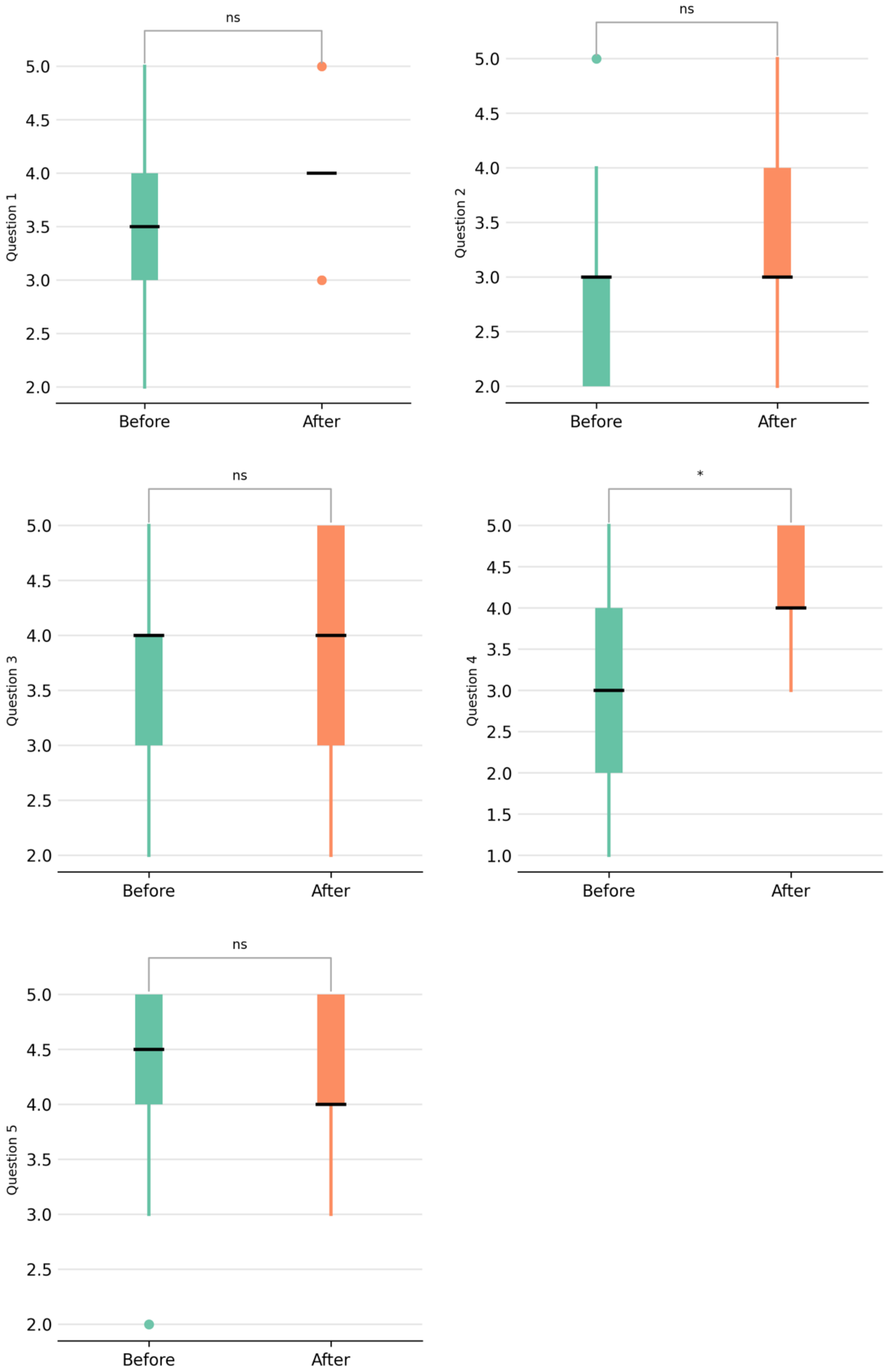

Supplementary Figure 5

Comparison between the teachers' responses in the Part B questions of the teacher's questionnaires before and after the BioSTEM session. ns: Not significant, *: p-value<0.05, **: p-value<0.01.

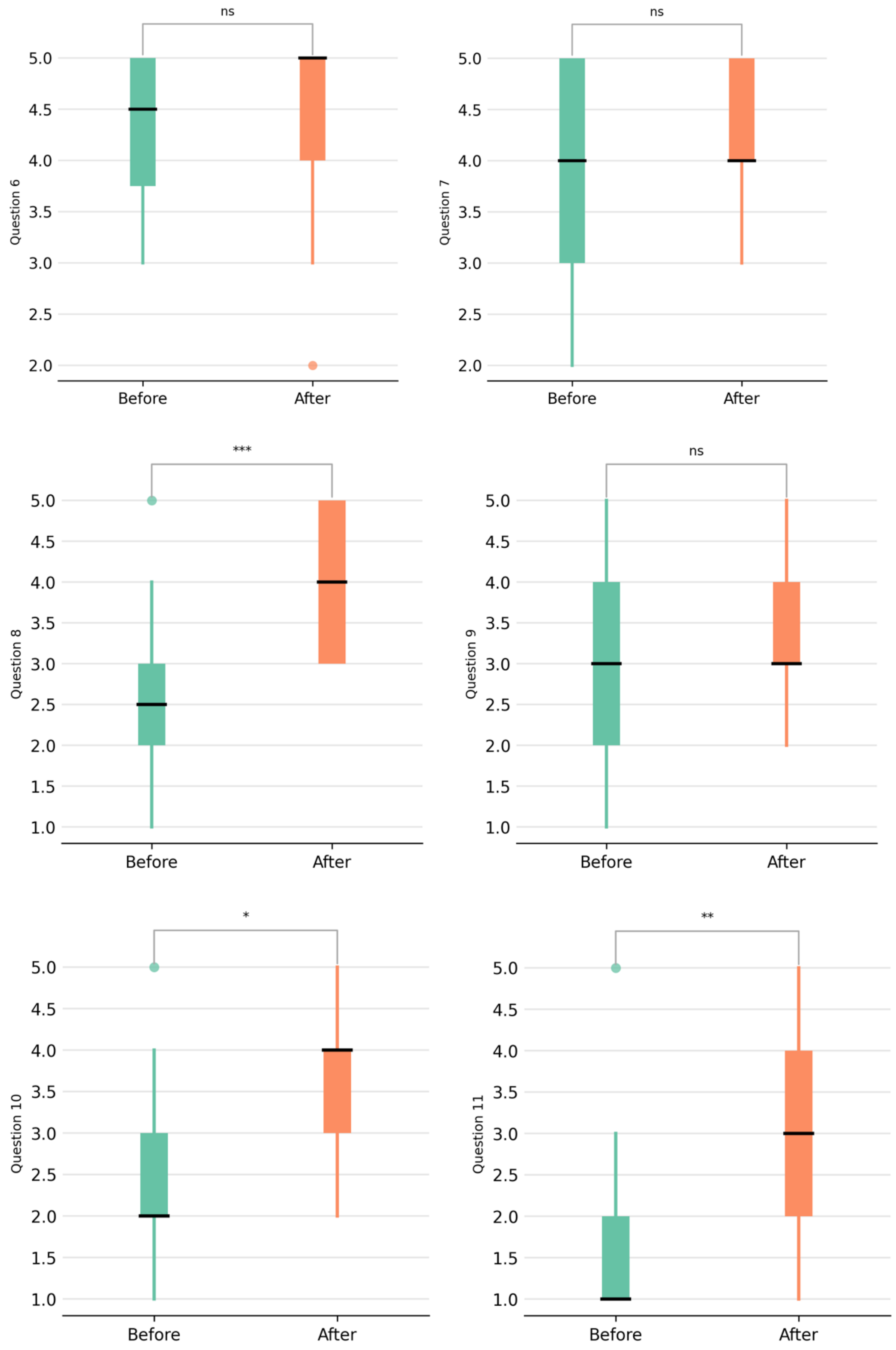

Supplementary Figure 6

Comparison between the teachers' responses in the Part C questions of the teacher's questionnaires before and after the BioSTEM session. ns: Not significant.

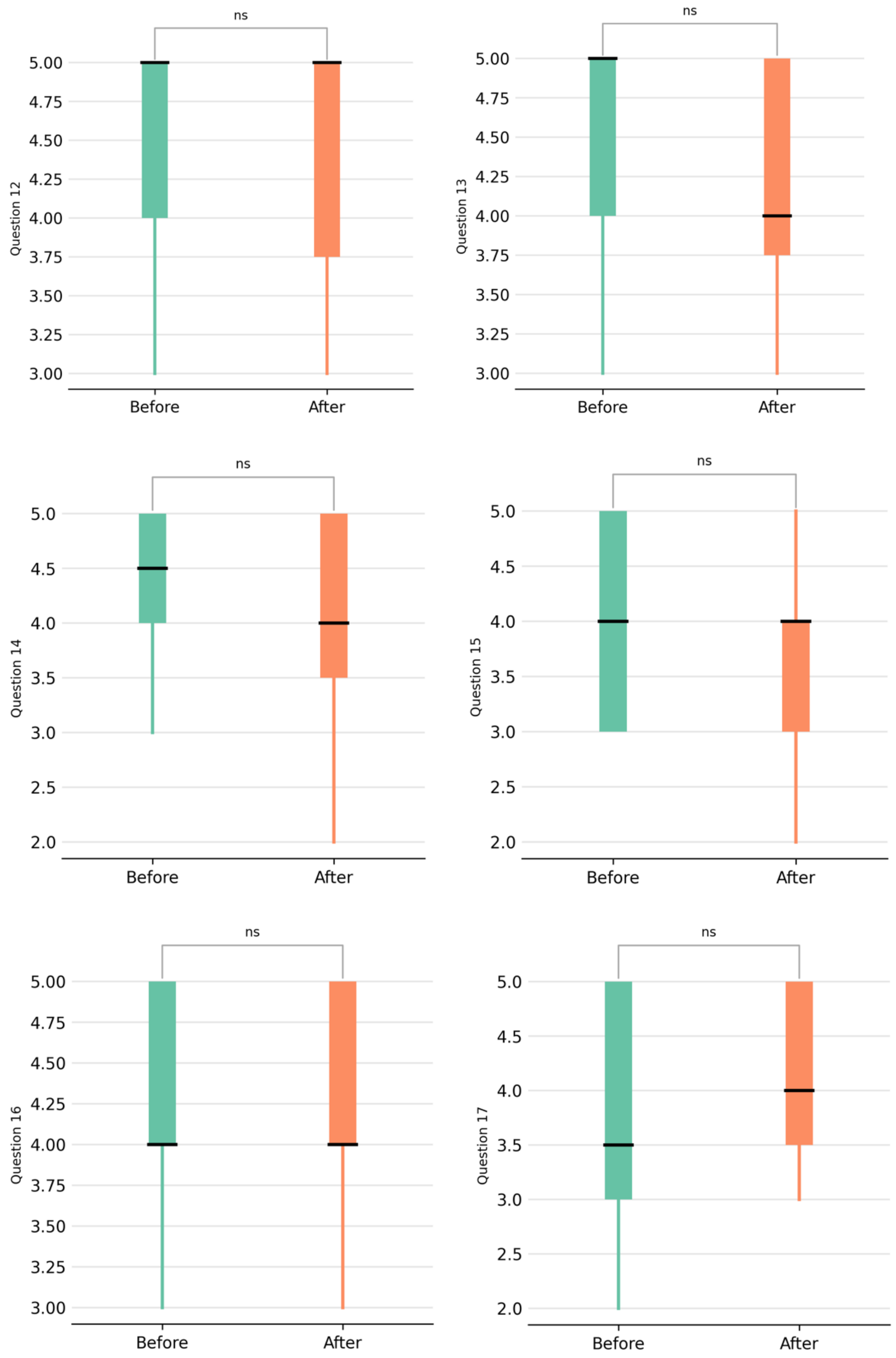

Supplement: Supplementary file 1 — Supplementary Material 1 [file 40246_2025_786_MOESM1_ESM.pdf]
